# Distribution, prevalence and intensity of moose nose bot fly (*Cephenemyia ulrichii*) larvae in moose (*Alces alces*) from Norway

**DOI:** 10.1016/j.ijppaw.2021.04.012

**Published:** 2021-04-27

**Authors:** Christer M. Rolandsen, Knut Madslien, Bjørnar Ytrehus, Inger Sofie Hamnes, Erling J. Solberg, Atle Mysterud, Turid Vikøren, Jørn Våge, Oddvar Hanssen, Andrea L. Miller

**Affiliations:** a Norwegian Institute for Nature Research (NINA), PO Box 5685 Sluppen, NO-7485, Trondheim, Norway; b Norwegian Veterinary Institute, P.O. Box 750 Sentrum, NO-0106, Oslo, Norway; c Centre for Ecological and Evolutionary Synthesis (CEES), Department of Biosciences, University of Oslo, P.O. Box 1066 Blindern, NO-0316, Oslo, Norway

**Keywords:** *Cephenemyia*, Parasites, Prevalence, Intensity, Distribution, Moose population density

## Abstract

High host density combined with climate change may lead to invasion of harmful parasites in cervid (host) populations. Bot flies (Diptera: Oestridae) are a group of ectoparasites that may have strong impact on their hosts, but data on the current distribution, prevalence and intensity of the moose nose bot fly (*Cephenemyia ulrichii*) in Scandinavia are lacking. We estimated prevalence and intensity of nose bot fly larvae in 30 moose from southern and 79 moose from central Norway. All larvae detected were identified as the moose nose bot fly. We found surprisingly high prevalence in these areas, which are up to 1300 km south-southwest of the first published location in Norway and west of the distribution in Sweden. Prevalence (0.44–1.00) was higher in areas with higher moose density. Parasite intensity in hunter killed moose was higher in central Norway (mean 5.7) than southern Norway (mean 2.9), and in both regions higher in calves and yearlings than adults. Fallen moose had higher parasite intensity (mean 9.8) compared to hunter killed moose in the subsample from central Norway, suggesting a link to host condition or behavior. Our study provides evidence of parasite range expansion, and establishing monitoring appears urgent to better understand impact on host populations.

## Highlights

• A marked geographic expansion of the moose nose bot fly in Norway is documented.
• We provide the first estimates of prevalence and intensity in Norwegian moose.
• Higher parasite prevalence was associated with higher moose density (host density).
• Higher parasite intensity in fallen than hunter killed moose suggest a link to host condition or behavior.

## Graphical abstract

**Figure:**
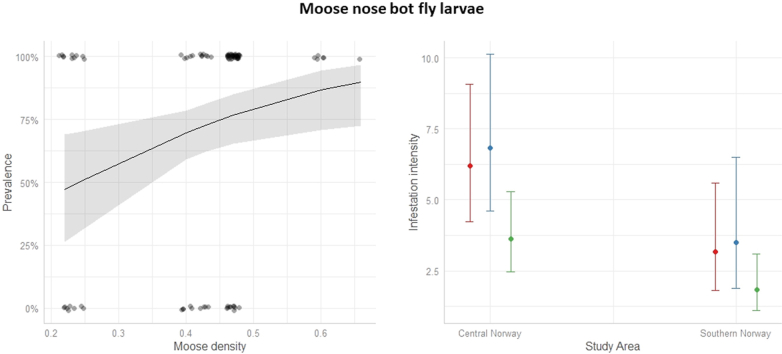

## 1 Introduction

Parasites influence the fitness of hosts through their effects on individual life-history components (Anderson and May 1978; Gulland, 1992; Irvine et al., 2006). Prevalence and intensity of parasites can increase with increasing host density (Body et al., 2011) and can negatively affect body weight and condition (Davidson et al., 2015; Hughes et al., 2009; Vicente et al., 2004). Level of parasitism may also vary with temperature, and there is increasing concern that the health of northern ungulates will deteriorate with changing climate (Kutz et al., 2005; Weladji et al., 2003). In the Fennoscandian countries, Norway, Sweden, and Finland, moose (*Alces alces*) densities have increased rapidly since the 1960s and are currently in general high (Lavsund et al., 2003), although varying among regions and countries. Both high host densities and climate warming can facilitate the expansion of parasite distribution ranges.

Bot flies, Diptera in the family Oestridae, are a group of ectoparasites with potential impact on the host as a result of (1) larviposition behavior of the adult females and (2) migration and development of larvae in their host. It has been suggested that the larviposition behavior of the nose bot flies in the genus *Cephenemyia* is similar, and that deer species can dramatically react to the larviposition behavior of these nose bot flies (Anderson, 1975). However, it has also been suggested that Oestridae larvae normally do not cause severe disease in otherwise healthy animals (Cogley, 1987; Colwell, 2001). For adult moose it is assumed that infestation with the moose nose bot fly (*Cephenemyia ulrichii*) larvae is rarely lethal, but that the general health condition may deteriorate markedly. For calves mortality may be higher when they are infested with a great number of larvae (Zumpt, 1965).

The moose nose bot fly is an obligate parasite with larvae inhabiting the nasal cavities and pharynx of moose. The female nose bot fly deposits or ejects packets of first instar (L1) larvae onto the nose region of the host whereupon the larvae move into the nose or mouth and migrate into the nasal cavities. Moose have complex nasopharyngeal cavities (Márquez et al., 2019) where the first, second (L2) and third (L3) instars can develop over the winter. By the spring, the L3 have migrated to the pharynx where they stay until the host ejects them out by coughing or sneezing, or they simply crawl out themselves. The larvae then pupate in the soil and hatch as flies during summer (Colwell, 2001). In Central Europe the flies swarm from the end of May until mid-September (Zumpt, 1965).

Data on the distribution and potential factors affecting bot fly infestation levels of moose are limited. The moose nose bot fly has been considered native to Central and Eastern Europe, but not to Scandinavia (Zumpt, 1965). In Norway, the first published documentation of the moose nose bot fly was in Pasvik in the northernmost part of Norway close to the Russian and Finnish border in 1987, but was in the same study not detected in other parts of northern or southern Norway (Nilssen and Haugerud, 1994). However, it appears from the Norwegian Veterinary Institute's (NVI) annual wildlife reports that moose nose bot fly larvae were found in a moose from Pasvik, suffering from bilateral cataracts (journal number 1985/80), already in 1980 (Holt and Stovner, 1980). It is likely that the parasite has expanded its distribution into Norway from the east or north as it was found in Finland in the early 1900s and in northern Sweden in the late 1970s and 1980s (Nilssen and Haugerud, 1994; Steen et al., 1988). One could theorize that the parasites then migrated southwards. This theory is supported by the detection of new locations for moose nose bot fly further south in Norway through passive health monitoring by the NVI; one moose in central Norway (Grong municipality) in 2003 (Lillehaug et al., 2004) and four moose further south (Røros, Kongsvinger and Oslo municipalities, between 60 and 63°N, 11 and 12°E) in 2014 (Handeland et al., 2015). To our knowledge no studies have quantified parasite prevalence of moose nose bot fly larvae, only presence in the host based on rather low sample sizes (e.g. N = 11, Steen et al., 1988), and there are no studies of the impact of this parasite on moose.

To better understand the distribution of the moose nose bot fly and the parasite's potential impact on their hosts, we here report the prevalence and intensity of the moose nose botfly in different sex and age groups of moose from two study areas in Norway. Based on moose shot during ordinary hunting, we explore whether parasite prevalence and parasite intensity are associated with moose density. We also explore whether parasite prevalence and intensity correlates with body mass in one of the regions, and contrast the prevalence and intensity of bot fly larvae in hunter killed moose and moose found dead for other reasons (fallen moose). In general, we predict parasite intensity to increase with host density, and to be higher in the sample of fallen moose than in moose shot during hunting. The latter prediction is based on the assumption that high parasite intensity decreases the condition of the host, or that sick or weakened hosts behave in a manner that makes them more prone to infestation by bot flies, and eventually die for reasons other than hunting.

## 2 Material and methods

### 2.1 Study area and collection of moose heads

**Fig. 1:**
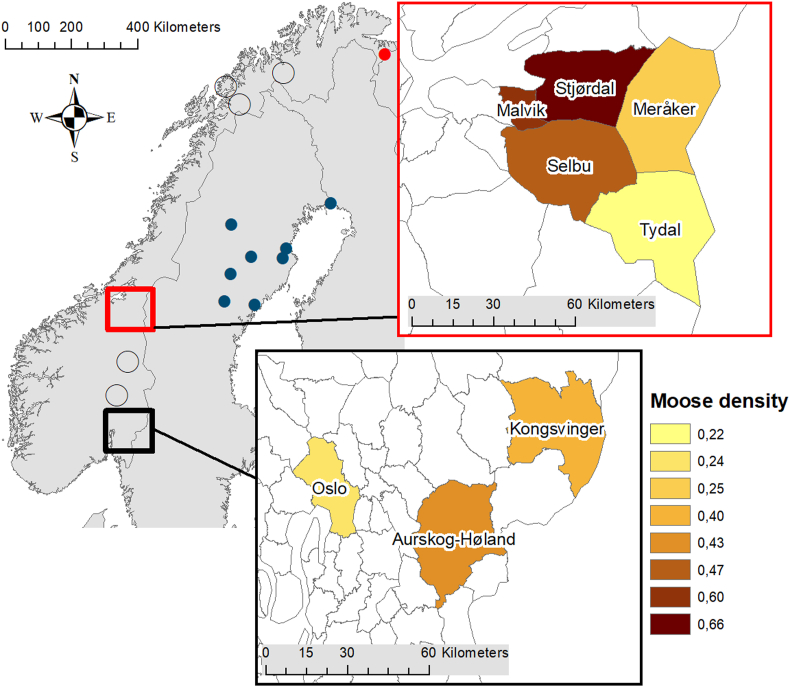
Study areas in southern (Oslo, Aurskog-Høland and Kongsvinger) and central Norway (Selbu, Tydal, Malvik, Stjørdal and Meråker) with location and moose density (moose density, see **Materials and methods**) in sampling municipalities. Red filled circle indicate where the moose nose bot fly (*Cephenemyia ulrichii*) was first found in Norway, and open circles show where moose heads were examined without detection of the moose nose bot fly in 1987 (Nilssen and Haugerud, 1994). Blue circles indicate where the moose nose bot fly were found in Sweden in the late 1970s and 1980s (Steen et al., 1988). (For interpretation of the references to colour in this figure legend, the reader is referred to the Web version of this article.)

We collected heads from 120 moose in eight municipalities: Oslo, Aurskog-Høland and Kongsvinger in southern Norway and Selbu, Tydal, Malvik, Stjørdal and Meråker in central Norway (Fig. 1). Data on summer temperature was available from weather stations (The Norwegian Meteorological Institute, www.met.no) in Oslo (met station no 18500, 360 m a.s.l.), Aurskog-Høland (met station no 2650, 128 m a.s.l.), Kongsvinger (met station no 5660, 170 m a.s.l.), Selbu (met station no 68290, 160 m a.s.l.), Stjørdal (met station no 69100, 12 m a.s.l.) and Meråker (met station no 69380, 169 m a.s.l.). For Tydal and Malvik there were no weather stations measuring temperature. The average of mean monthly temperature in June, July and August for these weather stations in the years moose heads were collected in each municipality varied between 12.6 °C and 14.1 °C (mean 13.3 °C ± 0.63 SD).

In southern Norway, 31 moose heads were collected in the period October 5–12 in 2015 from moose shot by hunters and examined by NVI as a part of the Norwegian health surveillance program for cervids and muskoxen (Madslien et al., 2017). One of these heads was excluded from further analysis because it was too damaged after the animal was shot in the neck/head by hunters. In central Norway, 89 moose heads were collected. Heads that were rotten or moderately damaged, particularly with partially or fully destroyed nasal cavities/sinuses, heads with too much food/rumen material in the nasal passages, or heads with missing identification data were eliminated from the study (n = 11). Due to the restricted sample size of heads from fallen moose, however, heads were included even if they were moderately damaged. In central Norway we included 62 moose killed by hunters in the period September 25 – November 18 in 2017, and 17 moose found dead due to vehicle collisions, other accidents or diseases (hereafter called fallen moose) in the period January 16 – August 17, 2018. These heads were collected as a part of an ongoing surveillance program for chronic wasting disease (CWD) and were examined by the Norwegian Institute for Nature Research (NINA) (e.g., Rolandsen et al., 2019).

As a proxy for population density, we used the number of harvested moose per km^2^ of forest and bogs (hereafter called moose density). This proxy has been tested against independent abundance data and usually provides an accurate reflection of the spatial variation in moose densities at the municipal scale (Ueno et al., 2014). We used the 3-year average of moose density in year t (year of death), t-1 and t-2 in each municipality as an index of spatial variation in density.

### 2.2 Parasite sampling and species identification

Heads collected from hunter killed moose were frozen at −20 °C until parasite analysis. Heads collected from fallen moose were often, but not always, frozen before analysis. All moose included from central Norway were confirmed CWD negative before analysis. Moose from southern Norway, however, were not tested for CWD, as these examinations were conducted in 2015, prior to the first detection of CWD in Norway in 2016 (Benestad et al., 2016; Pirisinu et al., 2018).

The parasite collection procedure was developed based on Nilssen and Haugerud (1995). After thawing, the heads were skinned and cut in half along the medial sagittal plane using a bandsaw. The nasal septum was removed, and the nasal cavities were grossly examined. Detected parasites were collected and stored in 70% ethanol immediately. Afterwards, the nasal cavities, sinuses, and pharynx were thoroughly washed using varying streams of water. The runoff was collected in a plastic tub. The ethmoid turbinate was then removed and washed into the same plastic tub. After loosening the internal structures of the nasal cavity (e.g. nostril connective tissue pad, *concha nasalis ventralis*, *plica recta* (Clifford and Witmer, 2004)) and opening the sinus cavities, the entire nasal cavity and *pharynx* was washed a second time. In moose from central Norway, a perforation was made from the outside through *os maxillare* to access *sinus maxillaris* (Clifford and Witmer, 2004), A stream of water was directed through this entrance to flush *sinus maxillaris* and connected sinuses (palatine, lacrimal) into the plastic tub. Each half of the head was examined separately by using the same procedure.

The water from the washings was sieved progressively through a sieve with a mesh size of 1 mm and a sieve with a mesh size of 250 μm (both 200 mm in diameter). Large debris were rinsed and removed from the 1 mm sieve. For moose in southern Norway, larvae were counted directly from each sieve. In central Norway, material remaining in each 200 mm diameter sieve was rinsed through a final sieve with a mesh size of 180 μm and diameter of 90 mm (Millipore, Billerica, Massachusetts). This final step was performed using a filter pump (Millipore, Billerica, Massachusetts). These smaller 180 μm sieves were removed and put into petri dishes for larval counting. If the larval counting could not be performed the same day, the contents were saved in the refrigerator (4 °C) until counting. Using a stereomicroscope, larvae were counted, identified and stored in 70% ethanol.

Parasites were identified morphologically based on Nilssen and Haugerud (1994) and Zumpt (1965). For the L1, differentiation of moose nose bot fly larvae from the closely related reindeer nose bot fly larvae was based mainly on the number of caudal hooks present (more than 14) and the rows of denticles present. For the L2 and L3, identification was based on presence of confirmed L1, spinulation of the body segments and posterior peritremes.

Parasite prevalence was defined as number of moose positive for one or more larvae divided by total number of moose examined. Parasite intensity was defined as the total number of larvae (all instar levels) per infected moose.

### 2.3 Statistical analysis

Prevalence has a binomial distribution and was analysed with logistic regression models in R (R, Core RCore Team and Computing, 2019). As explanatory variables we included main effects of study area, sex, age group (calf, yearling, adult), and moose population density. The separation into age groups was based on tooth eruption and replacement patterns. Spatial variation in moose population density was measured at the municipal scale. We also included the two-way interaction between sex and age group in the model selection. As temperature data was missing from two of eight municipalities and only measured at one location in other municipalities, we considered these data to be too coarse to add temperature as a spatial covariate.

Parasite intensity was analysed with zero-truncated GLM-models with negative binomial error distributions to account for overdispersion, in the R package countreg (Zeileis and Kleiber, 2016). Zero-truncated distributions were necessary as we only included infected individuals, i.e. parasite intensity could not equal zero. As for prevalence models we included study area, sex, age group, moose density, and the two-way interaction between sex and age group as explanatory variables.

For a subsample in the study area in central Norway we had data on carcass mass (approximately 50% of live body mass (Langvatn, 1977) and exact age in years (Rolandsen et al., 2008; Veiberg et al., 2020) of adults. Because carcass mass of calves tends to increase during the fall hunting season (Tiilikainen et al., 2012), we adjusted all masses to October 15 by using a regression of carcass mass on kill date. However, both adjusted and original masses produced qualitatively similar results. We added body mass and exact age as explanatory variables to the highest ranked prevalence and intensity models from central Norway. We tried models with body mass and log(body mass), as log transformation can make skewed distributions less skewed, reduce potential effects of outliers and make data easier to interpret. For the same region we also performed a separate analysis with data source (hunted, fallen moose) included as a factor. For fallen moose we did not have data on body mass and exact age, but included sex, age group, and moose density.

We performed model selection based on the Akaike's information criterion (AIC) corrected for small sample size (AICc; Burnham and Anderson, 2002), and in addition to the highest ranked model we report models within ΔAIC_c_ ≤ 2 from that model. In supplementary tables we report ΔAIC_c_ and Akaike weights from all combinations of covariates based on automated model selection using the R package MuMIn (Barton, 2009). Figures were made with R packages ggplot2 (Wickham, 2016) and ggeffects (Lüdecke, 2018).

## 3 Results

We identified only moose nose bot fly larvae (*C. ulrichii*), and overall parasite prevalence was 0.72 (n = 109), while overall parasite intensity of infected moose averaged 5.8 larvae per individual (range 1–28 larvae) (Table 1).

**Table 1:** Number of moose heads examined for nose bot fly larvae in total and distributed by sex, age group and study area in Norway. In Central Norway, numbers are given for each data source (hunter killed moose and fallen moose). N_tot=_total number of moose examined, N_inf_ = number of infected moose. Prevalence (Prev.) = N_inf_/N_tot_. For infected moose, the mean intensity, median intensity and minimum (min.) and maximum (max.) number of larvae are given.

Study area	Age group	N_tot_	N_inf_	Prev.		Intensity		
					Mean	Median	Min.	Max.
Total	All	109	78	0.72	5.8	4.0	1	28
	Calves	30	23	0.77	5.8	5.0	1	17
	Yearlings	25	17	0.68	7.4	6.0	1	17
	Adults	54	38	0.70	5.1	4.0	1	28

Southern Norway,	All	30	18	0.60	2.9	2.0	1	9
hunted	Calves	6	3	0.50	6.3	7.0	3	9
	Yearlings	3	3	1.00	2.0	2.0	2	2
	Adults	21	12	0.57	2.3	2.0	1	5

Central Norway,	All	62	47	0.76	5.7	4.0	1	17
hunted	Calves	20	16	0.8	5.8	5.0	1	17
	Yearlings	20	13	0.65	7.8	9.0	1	16
	Adults	22	18	0.82	4.2	4.0	2	8

Central Norway,	All	17	13	0.76	9.8	8.0	1	28
fallen stock	Calves	4	4	1.00	5.5	5.5	2	9
	Yearlings	2	1	0.50	17.0	17.0	17	17
	Adults	11	8	0.73	11.1	8.5	1	28

All larvae from hunter killed moose were identified as L1, except for one L3 (38 mm) that was found trapped in the *sinus maxillaris* of a moose shot in September 2017. Larvae found in hunter killed moose in central Norway ranged from 1.6 to 3.4 mm in length. In most fallen moose collected in January, February, March and August, larvae ranged in size from 1.5 to 3.9 mm. However, one fallen moose collected in February and two fallen moose collected in March contained larvae with sizes reaching a maximum of 5.2 mm, 5.7 mm and 11.2 mm respectively. These larvae were presumed to be transitioning from L1 to L2.

In the subsample of moose shot during hunting in central Norway, the mean (±SD) carcass mass was 61.9 kg (±10.0), 116.1 kg (±20.2) and 151.9 kg (±24.8), for calves, yearling and adults, respectively, and the age of adult moose (≥2 years) varied from 2 to 8 years (3.5 ± 1.8 SD). Moose density varied between 0.22 and 0.66 (mean 0.41 ± 0.17 SD).

### 3.1 Prevalence and intensity in moose shot during hunting

**Fig. 2:**
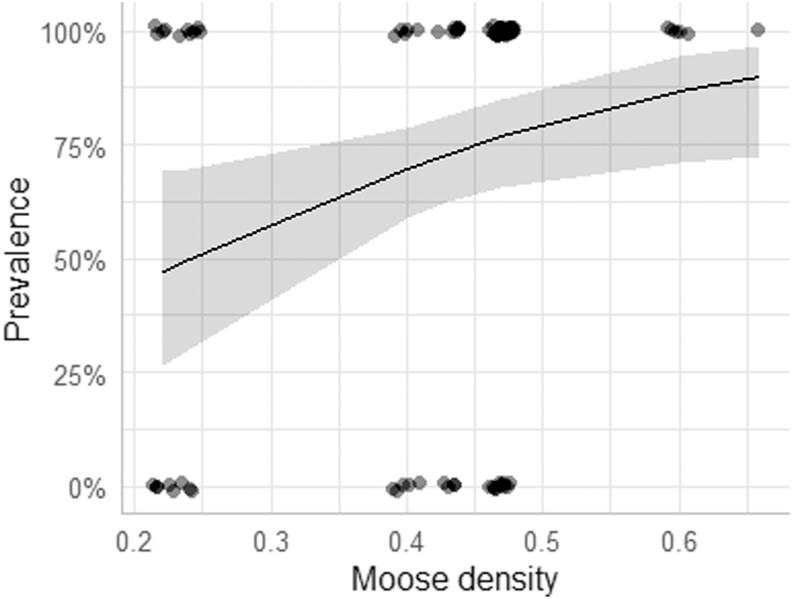
The predicted infection prevalence of moose nose bot fly larvae with increasing moose (host) density. The shaded area shows the 95% confidence interval. Predictions from the highest ranked model with moose density. In the plot we used the function “jitter” in the R package ggeffects (Lüdecke, 2018), which adds small random variation to the data points to better reflect the amount of data for moose densities. Hence, the points do not reflect exact values as they are binomial.

The highest ranked prevalence model included a positive effect of moose density (AIC_c_ = 109.82, Fig. 2, β = 5.205, SE = 2.221, Z = 2.34, P = 0.019). The effect of moose density remained significant (P = 0.049) after controlling for study area in an alternative model (ΔAIC_c_ = 1.57) and was significant (P = 0.014) if we only used data from central Norway. An alternative model with moose density and sex was marginally competitive based on AIC (ΔAIC_c_ = 1.97), but the parameter estimate for sex suggesting higher prevalence in males compared to females, was highly uncertain when estimated (P = 0.68). Other models had weaker support (ΔAIC_c_ > 2, Supplementary Table S1).

**Fig. 3:**
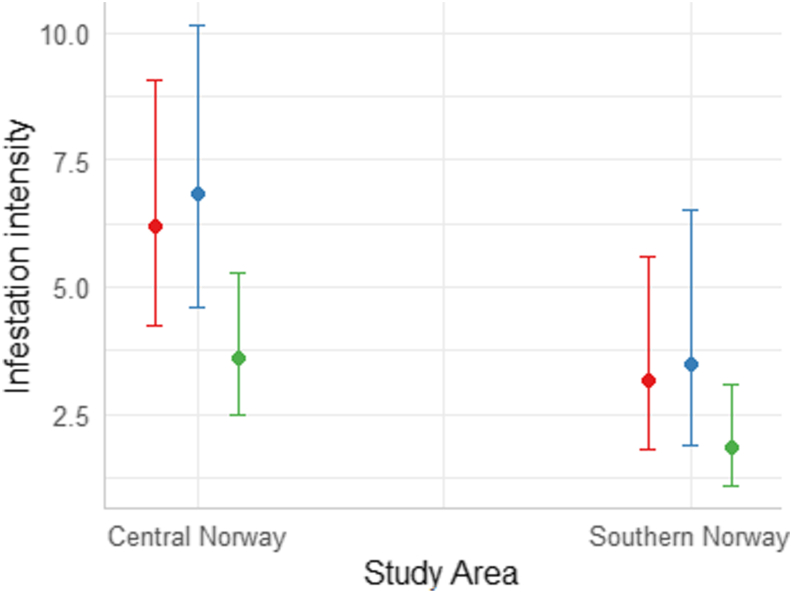
The predicted parasite intensity of moose nose bot fly larvae for harvested calves (red), yearlings (blue) and adult (green) moose in central and southern Norway. Predictions from the highest ranked intensity model with study area and age group. (For interpretation of the references to colour in this figure legend, the reader is referred to the Web version of this article.)

The highest ranked intensity model included study area (β = −0.671, SE = 0.270, Z = −2.492, P = 0.013) and age group (Adults-Calves: β = −0.541, SE = 0.255, Z = −2.118, P = 0.034, Yearlings-Calves: β = 0.096, SE = 0.272, Z = 0.354, P = 0.723) (AIC_c_ = 319.66), predicting that moose in central Norway were infested with approximately twice as many larvae as moose in southern Norway (6.2 vs. 3.1, 6.8 vs. 3.5 and 3.6 vs. 1.8, in calves, yearlings and adults, respectively, Fig. 3). Models including sex or moose density had weaker support (ΔAIC_c_ > 2, Supplementary Table S2).

Adding body mass or log(body mass) and the interaction between age group and body mass while controlling for sex did not improve the highest ranked prevalence model with density in central Norway (ΔAIC_c_ > 2, Supplementary Table S3), and the same was true when adding exact age as a covariate or factor instead of age group (ΔAIC_c_ > 2).

### 3.2 Prevalence and intensity in fallen moose versus hunter killed moose

Adding data source (moose shot during hunting vs. fallen moose) did not improve the parasite prevalence model in central Norway (ΔAIC_c_ > 2). Analyzing the parasite prevalence without moose density returned the intercept-only model as the highest ranked (AICc = 89.21), while models including data source, sex and age group had weaker support (ΔAIC_c_ > 2).

In central Norway, the highest ranked intensity model included data source (β = −0.597, SE = 0.277, Z = −2.159, P = 0.031, AIC_c_ = 338.52), predicting that parasite intensity was higher in fallen moose (9.4, 95% CI [5.8,15.1]) than in hunter killed moose (5.2, 95% CI [3.9,6.8]). This effect remained significant (P = 0.008 and P = 0.030) in alternative models including main effects of age group (ΔAIC_c=_0.30) or moose density (ΔAIC_c=_2.01), respectively. Models including sex or the interaction between sex and age had weaker support (ΔAIC_c_ > 2).

## 4 Discussion

In the face of global warming there is a growing concern that many parasites will expand and increase their impact on wild cervids in the northern hemisphere. We show that the moose nose bot fly is now present with high prevalence in moose in central and southern Norway, approximately 1300 km south-southwest of the first published location in Pasvik, Norway (Nilssen and Haugerud, 1994), and west of the distribution in Sweden (Jaenson, 2011; Steen et al., 1988). This indicates a considerable range expansion of the moose nose bot fly in Fennoscandia. Being the first to estimate prevalence and intensity of moose nose bot fly larvae in their host, we also document markedly higher prevalence in areas with high moose density, possibly adding to the adverse density effects on moose body condition seen in some of these populations (Solberg et al., 2017).

### 4.1 Distribution range expansion, host density and climate

Parasite geographic ranges depend on the interplay between host density and climate, of which the latter is mainly related to the timing and duration of their off-host periods. Another ectoparasite, the deer ked (*Lipoptena cervi*), has been invading Fennoscandian moose populations from two directions in recent decades (Valimaki et al., 2010). In Norway and Sweden, range invasion has been from the south, while another variant of deer ked has colonized southern and central Finland from Russia. Interestingly, the moose bot fly seems to have colonized in the reverse direction, from north to south in Norway. The deer ked has an off-host stage over-winter as pupae that may be prone to climate limitation of the distribution (Valimaki et al., 2010), while the flight activity and development of bot flies is also temperature dependent (Anderson et al., 1994). Currently, we have no strong evidence of the relative effect of density and climate on the expanding distribution of these parasites. The available temperature data in our study was too coarse to conduct a meaningful analysis of the effect of temperature on moose bot fly infestation levels. Such analysis would require data with more detailed spatial resolution, and preferably also temporal data to be able to analyze year-to-year variation. We also had low variation in summer temperature between study areas. A previous study of the closely related reindeer nose bot fly found higher infestation levels in reindeer (*Rangifer tarandus*) in warmer than colder summers (Nilssen and Haugerud, 1994), which was explained by the fact that the transmission rate is directly dependent on the summer weather. In reindeer nose bot fly, the lower threshold temperature for flying has been found to be within 13–15 °C (Breyev, 1956; 1961, as cited in Nilssen and Haugerud 1995), but whether this threshold also applies for the moose nose bot fly is to our knowledge not known.

Host specificity may also affect range expansion. The deer ked invading Finland appears host specific to moose and range expansion seems to end in the reindeer areas in the north, while the variant expanding from south in Norway and Sweden appears to infest at least roe deer, red deer (*Cervus elaphus*) and moose. Another ectoparasite, the *Ixodes ricinus* tick, is a host generalist and is increasing in elevational range in continental Europe (Medlock et al., 2013) and latitudinal range in Scandinavia (Jaenson and Lindgren, 2011; Jore et al., 2014) over a wide range of host communities, probably linked to global warming. The moose nose bot fly is thought to be host specific (Zumpt, 1965), but is once reported in roe deer in Finland (Nilssen et al., 2008), and is also found to cause (rarely) myasis in humans (Jaenson, 2011). In Fennoscandia, only the moose nose bot fly has been found in moose, while the reindeer nose bot fly is common in reindeer (Nilssen and Haugerud, 1995). We identified only moose nose bot fly larvae in moose, and moose is likely the most important host driving the moose nose bot fly distribution in Norway. However, until several potential host species inhabiting the same areas are examined we think it is immature to firmly conclude about the host specificity of the moose nose bot fly in Norway. The roe deer nose bot fly was reported in Sweden for the first time in 2012 (Molander, 2013), while other nose bot flies found in Europe, such as *C. auribarbis* host specific to red deer or fallow deer (*Dama dama*), or *Pharyngomyia picta* host specific to red deer, roe deer and fallow deer (Colwell, 2001), have not been detected in Fennoscandia.

### 4.2 Timing since parasite introduction

From our and previous studies (e.g. Dudzinski, 1970) the effect of host density on nose bot fly prevalence seems quite clear; however, based on currently available data we don't know to what degree the geographical distribution is also linked to time since the parasite was introduced. The first study documenting moose nose bot fly in Norway found 6 L1 larvae in one of three examined moose in the northernmost region, while none were found in a total of 20 moose from five other regions (Nilssen and Haugerud, 1994, see also Fig. 1). In our study we found moose nose bot fly larvae in all eight municipalities. Two of the municipalities included in Nilssen and Haugerud (1994) were in southern Norway, located between our study areas in central and southern Norway (Fig. 1). This may suggest that the moose nose bot fly more recently have established in southern than central Norway or further north and east, although the small samples in the two southern municipalities (N = 5 and N = 2) in Nilssen and Haugerud (1994) precludes any strong conclusion. In support of such a claim, however, we found a study area effect in addition to the effect of host density, indicating higher prevalence and intensity in central Norway compared with southern Norway. This is further supported by the fact that passive health monitoring of cervids found the parasite in moose in central Norway (Grong municipality) in 2003 (Lillehaug et al., 2004) and in southern Norway (Røros, Kongsvinger and Oslo municipalities) 11 years later (2014) (Handeland et al., 2015). We also acknowledge that the effect of study area may possibly have been caused by two different laboratories sampling the parasites, but the differences in methods was minor and not expected to be the major cause of the observed difference. However, the difference could also have been due to a variation between years as the moose heads were collected in different years. Therefore, a further investigation of the potential effect of timing since parasite introduction require more data.

### 4.3 Evidence of age and sex differences and effect on hosts

Moose as a species seems to become more vulnerable to parasites under climate change, as evidenced from their southern distribution ranges in North America (Escobar et al., 2019; Murray et al., 2006). Therefore, the expansion of the moose bot fly is of interest for moose population performance, also in combination with other coinfecting parasites. We assessed potential effects of moose nose bot fly on hosts based on (1) correlation to population density across regions, (2) differences between sex and age groups, (3) body mass within a region, and (4) comparing fallen moose with moose shot during hunting. Bot fly larvae from *Cephenemyia* spp. has been reported to incidentally cause death to black-tailed deer (*Odocoileus hemionus*) (Walker, 1929), may cause severe pathologic changes (Cogley, 1987), and can markedly reduce the health condition of moose and other ungulates (Zumpt, 1965). The sheep nose bot fly (*Oestrus ovis*) is known to cause weight loss in domestic sheep (*Ovis aries*) (El-Tahawy, 2010). The infection may cause tissue lesions resulting from the mechanical action of spines and hooks during larval movement on mucosal membranes, and, at least in sheep and goat, pronounced inflammatory response of the host (Angulo-Valadez et al., 2011). The combined insect harassment of the reindeer nose bot fly and the reindeer warble fly (*Hypoderma tarandi*), which can have several potential negative effects including reduced grazing time, has been linked to reduced autumn weight of reindeer calves in Norway (Weladji et al., 2003). We found increased parasite load in areas with higher moose density, higher parasite intensity in calves and yearlings than adults, and in fallen moose compared to hunter killed moose. This suggests that high moose density can increase the impact of the moose nose bot fly, and probably more profoundly in calves and yearlings than adults. Similarly, increasing parasitism by the winter tick (*Dermacentor albipictus*), facilitated by high host (moose) density, is reported to negatively affect moose survival and fecundity in northeast USA, which has led researchers to suggest a shift in moose management strategy focused on lowering moose density (Ellingwood et al., 2020).

We found no convincing effect of moose sex in our study. A lower ranked prevalence model (ΔAIC_c_ = 1.97) included sex, but with a highly uncertain (P = 0.68) parameter estimate suggesting no difference in prevalence of bot fly larvae between male and female moose in our study. Higher parasite prevalence and intensity in males than in females have been found in roe deer (e.g. Dudzinski, 1970).

The higher parasite intensity in fallen moose than moose shot during hunting suggests a possible link to host condition or behavior. Infection of bot flies (*C. auribarbis and P. picta)* was associated with lower body condition of young red deer in Spain (Vicente et al., 2004), but not for bot flies (*C. stimulator*) in roe deer in the Czech Republic (Salaba et al., 2013). We did not find an effect of body mass on the probability of infection when controlling for moose density in our subset with data from central Norway. More data is needed to explore whether parasite load may be linked to body mass or other proxies for individual condition of the host, while simultaneously controlling for effects of host density on host condition. Moreover, the effect of bot flies on hosts is not necessarily only a function of parasite intensity, but also related to the immune response, including that immunotolerance may occur after repeated infections (Angulo-Valadez et al., 2011; Jacquiet et al., 2005).

### 4.4 Larvae development

The development period of L1 for different species of bot flies are in general considered to vary widely, ranging from several days to several months. The larvae can vary in length, and factors affecting L1 development are not clearly understood, although there are indications that larval crowding and host immunity are components in the regulatory process (Colwell, 2001). Our measures of minimum and maximum size of larvae did not show any increasing trend from September until March for L1. For L2 and L3 we did not have data to analyze size development. In future studies, all larvae should therefore be measured individually to enable a better evaluation of size development.

L2 and L3 development often takes place at different sites in the nasal cavities or throats of hosts and can be quite variable in length (Colwell, 2001). We did not examine this in detail, but more detailed studies of the anatomical location of different larval stages and pathological lesions may increase the understanding of effects on hosts. We found one L3 trapped in the *sinus maxillaris* of a moose hunted in September 2017. This is likely a larva that was trapped over summer and did not drop from the host the preceding summer. This has also been found in the reindeer nose bot fly infecting reindeer in Norway (Nilssen and Haugerud, 1995).

## 5 Conclusion

Our study has increased the knowledge about the distribution of the moose nose bot fly in Norway and provides the first estimates of parasite prevalence and parasite intensity since the first discovery in the 1980s (Holt and Stovner, 1980; Nilssen and Haugerud, 1994). We also show that higher parasite prevalence is associated with higher moose density (host density), while we found no support for an effect of body mass. To more comprehensively understand the distribution pattern and potential effects of the moose nose bot fly on their host, we need more studies of parasite prevalence, parasite intensity and pathology, as well as how this may be associated with host density, body mass, age and sex, immune responses and climatic factors. As of now, there is no regular, active monitoring of parasites of cervids in the same region that can help to document effects on hosts, and the geographic coverage of current sampling is often limited. By documenting a marked geographic expansion of the moose nose bot fly, our study provides arguments for more comprehensive monitoring as a basis for future evidence-based management of cervids in Scandinavia and elsewhere. We also encourage studies of bot flies as potential vectors of pathogens. Pathogens have been detected in bot fly larvae (Scheid et al., 2016), and have in other insects survived metamorphosis (Duneau and Lazzaro, 2018). Indeed, myiasis-inducing flies in the family Oestridae have also been hypothesized to be one of several groups of ectoparasites that can transmit CWD (Lupi, 2005), but as far as we know this hypothesis has not been tested.

## Declaration of competing interest

None.
