# Supplementary material for: Distribution, prevalence and intensity of moose nose bot fly (Cephenemyia ulrichii) larvae in moose (Alces alces) from Norway
Source: Int J Parasitol Parasites Wildl. 2021 Apr 27;15:120–6. doi: 10.1016/j.ijppaw.2021.04.012 (PMC8105593; doi:10.1016/j.ijppaw.2021.04.012)
Supplement: Multimedia component 1 [file mmc1.docx]

**Supplementary material**

**Table S1.** Model selection table for the effect of study area, sex, age group and moose density, and the two-way interaction between sex and age group on parasite prevalence. The models are shown in decreasing order with the highest ranked model first.

| Model | Moose density | Study area | Age group | Sex | Sex:Age group | AICc | Delta AICc | AICc weight |
| --- | --- | --- | --- | --- | --- | --- | --- | --- |
| 1 | x |  |  |  |  | 109.82 | 0,00 | 0.34 |
| 2 | x | x |  |  |  | 111.39 | 1,57 | 0.15 |
| 3 | x |  |  | x |  | 111.79 | 1,97 | 0.13 |
| 4 |  | x |  |  |  | 113.12 | 3,3 | 0.06 |
| 5 | x | x |  | x |  | 113.39 | 3,57 | 0.06 |
| 6* |  |  |  |  |  | 113.41 | 3,59 | 0.06 |
| 7 | x |  | x |  |  | 113.74 | 3,92 | 0.05 |
| 8 |  | x |  | x |  | 115.13 | 5,31 | 0.02 |
| 9 | x |  | x | x | x | 115.23 | 5,41 | 0.02 |
| 10 | x | x | x |  |  | 115.4 | 5,58 | 0.02 |
| 11 |  |  |  | x |  | 115.42 | 5,6 | 0.02 |
| 12 | x |  | x | x |  | 115.85 | 6,03 | 0.02 |
| 13 | x | x | x | x | x | 116.72 | 6,9 | 0.01 |
| 14 |  | x | x | x | x | 117.09 | 7,26 | 0.01 |
| 15 |  | x | x |  |  | 117.15 | 7,33 | 0.01 |
| 16 |  |  | x |  |  | 117.53 | 7,71 | 0.01 |
| 17 | x | x | x | x |  | 117.54 | 7,72 | 0.01 |
| 18 |  |  | x | x | x | 117.69 | 7,87 | 0.01 |
| 19 |  | x | x | x |  | 119.26 | 9,44 | 0 |
| 20 |  |  | x | x |  | 119.65 | 9,83 | 0 |

**Intercept-only model*

**Table S2.** Model selection table for the effect of study area, sex, age group, moose density, summer temperature, and the two-way interaction between sex and age group on parasite intensity. The models are shown in decreasing order with the highest ranked model first.

| Model | Moose density | Study area | Age group | Sex | Sex:Age group | AICc | Delta AICc | AICc weight |
| --- | --- | --- | --- | --- | --- | --- | --- | --- |
| 1 |  | x | x |  |  | 319.66 | 0 | 0.34 |
| 2 |  | x |  |  |  | 321.72 | 2.06 | 0.12 |
| 3 | x | x | x |  |  | 321.93 | 2.27 | 0.11 |
| 4 |  | x | x | x |  | 322.03 | 2.36 | 0.10 |
| 5 |  | x |  | x |  | 323.31 | 3.65 | 0.06 |
| 6 |  |  | x |  |  | 323.32 | 3.66 | 0.05 |
| 7 | x | x |  |  |  | 323.51 | 3.85 | 0.05 |
| 8 |  | x | x | x | x | 324.07 | 4.41 | 0.04 |
| 9 | x | x | x | x |  | 324.4 | 4.74 | 0.03 |
| 10 | x |  | x |  |  | 325.06 | 5.39 | 0.02 |
| 11 | x | x |  | x |  | 325.34 | 5.67 | 0.02 |
| 12 |  |  | x | x |  | 325.62 | 5.96 | 0.02 |
| 13 | x | x | x | x | x | 326.77 | 7.1 | 0.01 |
| 14 | x |  | x | x |  | 327.39 | 7.73 | 0.01 |
| 15* |  |  |  |  |  | 327.96 | 8.3 | 0.01 |
| 16 |  |  | x | x | x | 328.6 | 8.93 | 0 |
| 17 |  |  |  | x |  | 329.44 | 9.78 | 0 |
| 18 | x |  |  |  |  | 329.68 | 10.02 | 0 |
| 19 | x |  | x | x | x | 329.81 | 10.15 | 0 |
| 20 | x |  |  | x |  | 331.04 | 11.38 | 0 |

**Intercept-only model*

**Table S3.** Comparison of change in AICc and AICs weights when adding body mass while controlling for sex and age group (2 and 3), to the highest ranked prevalence model with moose density (1). This was based on a subsample from central Norway with data on body mass and/or exact age. Using log(body mass) instead of body mass and/or exact age in years instead of age group gave qualitatively similar results.

| **Model** | **Moose density** | **Age group** | **Sex** | **Body mass** | **AICc** | **Delta AICc** | **AICc weight** |
| --- | --- | --- | --- | --- | --- | --- | --- |
| 1 | x |  |  |  | 58.82 | 0.00 | 0.91 |
| 2 | x | x | x |  | 63.79 | 4.97 | 0.08 |
| 3 | x | x | x | x | 66.77 | 7.95 | 0.02 |
